# Diethyl 2-(Phenylcarbamoyl)phenyl Phosphorothioates: Synthesis, Antimycobacterial Activity and Cholinesterase Inhibition

**DOI:** 10.3390/molecules19067152

**Published:** 2014-05-30

**Authors:** Jarmila Vinšová, Martin Krátký, Markéta Komlóová, Echchukattula Dadapeer, Šárka Štěpánková, Katarína Vorčáková, Jiřina Stolaříková

**Affiliations:** 1 Department of Inorganic and Organic Chemistry, Faculty of Pharmacy, Charles University, Heyrovského 1203, 500 05 Hradec Králové, Czech Republic; E-Mails: martin.kratky@faf.cuni.cz (M.K.); marketa.komloova@faf.cuni.cz (M.K.); dadapeer78@gmail.com (E.D.); 2 Department of Biological and Biochemical Sciences, Faculty of Chemical Technology, University of Pardubice, Studentská 573, 532 10 Pardubice, Czech Republic; E-Mails: sarka.stepankova@upce.cz (Š.Š.); katarina.vorcakova@student.upce.cz (K.V.); 3 Laboratory for Mycobacterial Diagnostics and Tuberculosis, Regional Institute of Public Health in Ostrava, Partyzánské náměstí 7, 702 00 Ostrava, Czech Republic; E-Mail: Jirina.Stolarikova@zu.cz

**Keywords:** antimycobacterial activity, *in vitro* acetylcholinesterase inhibition, *in vitro* butyrylcholinesteraseinhibition, salicylanilide, thiophosphates

## Abstract

A new series of 27 diethyl 2-(phenylcarbamoyl)phenyl phosphorothioates (thiophosphates) was synthesized, characterized by NMR, IR and CHN analyses and evaluated against *Mycobacterium tuberculosis* H_37_Rv, *Mycobacterium avium* and two strains of *Mycobacterium kansasii*. The best activity against *M. tuberculosis* was found for *O*-{4-bromo-2-[(3,4-dichlorophenyl)carbamoyl]phenyl} *O*,*O*-diethyl phosphorothioate (minimum inhibitory concentration of 4 µM). The highest activity against nontuberculous mycobacteria was exhibited by *O*-(5-chloro-2-{[4-(trifluoromethyl)phenyl]carbamoyl}-phenyl) *O*,*O*-diethyl phosphorothioate with MIC values from 16 µM. Prepared thiophosphates were also evaluated against acetylcholinesterase from electric eel and butyrylcholinesterase from equine serum. Their inhibitory activity was compared to that of the known cholinesterases inhibitors galanthamine and rivastigmine. All tested compounds showed a higher (for AChE inhibition) and comparable (for BChE inhibition) activity to that of rivastigmine, with IC_50_s within the 8.04 to 20.2 µM range.

## 1. Introduction

Tuberculosis (TB) is an infectious disease caused by intracellular pathogen *Mycobacterium tuberculosis* (*Mtb*). In the past, the occurrence of TB decreased thanks to the progress in medical care and therapeutic options, but nowadays, affecting more than eight million people worldwide, TB is once again considered to be a global health problem. In 2012, 1.3 million people died from the disease [1]. The rise of multidrug-resistant TB (MDR-TB) and extensively drug-resistant TB (XDR-TB) cases and the co-infection with HIV are the main reasons for its recurrence. Demanding pharmacological treatment and urgent need for new antimycobacterial agents present serious challenges, stimulating research in this area. 

A series of salicylanilide diethyl phosphates, previously synthesized by our group [2], showed an increased antimycobacterial activity, a decreased cytotoxicity and improved selectivity indices in comparison with the parent salicylanilides. Some of the compounds were also found to inhibit multidrug-resistant *Mtb* strains with a minimum inhibitory concentration (MIC) of 1 µM. We have concluded that temporary masking of the free hydroxyl group of salicylanilide compounds may increase/be beneficial for its possible usage and the formation of this kind of prodrug is an attractive strategy for an efficient cellular internalization [2].

In previous studies, the synthesis of thiophosphates has been involved in the synthesis of potential immunotherapeutic oligonucleotide prodrugs [3], thermolytic DNA prodrugs [4] and the synthesis of thiophosphate esters of 2'-C-methyl ribonucleosides used as inhibitors of RNA-dependent RNA polymerase of hepatitis C virus [5]. This structure modification served as an inspiration for the incorporation of sulphur into the phosphate moiety and preparation of thioanalogues with increased lipophilicity—*O*,*O*-diethyl *O*-[2-(phenylcarbamoyl)phenyl] phosphorothioates (salicylanilide diethyl thiophosphates).

Compounds with phosphonic, phosphoric or thiophosphoric moieties in their structures are well-known anticholinesterase agents. They act as irreversible inhibitors of both acetylcholinesterase (AChE, EC 3.1.1.7) and butyrylcholinesterase (BChE, EC 3.1.1.8) [6]. The role of AChE in the cholinergic synapsis is the termination of cholinergic impulse by hydrolysis of acetylcholine (ACh), a neurotransmitter present in central and peripheral nervous system [7]. With the inhibition of AChE, the level of ACh in the synaptic junction increases, enhancing thus cholinergic transmission. Reversible inhibitors of AChE are used in the treatment of various diseases, requiring a longer duration of ACh action on postsynaptic receptors (e.g., myasthenia gravis, Alzheimer’s disease) [8,9]. However, the irreversible inhibition by organophosphorus compounds can graduate into cholinergic crisis leading to overstimulation of cholinergic receptors which can be life-threatening. In pathological states accompanied by AChE depletion, BChE can partially compensate its regulatory function. Lacking the high specificity for the substrate, BChE is also a very important detoxicating protein, acting as a scavenger of esteratic exogenous compounds. However, its principal role in the organism still remains unclear [10]. Organophosphorus irreversible inhibitors express a strong inhibitory activity of both AChE and BChE, resulting from the formation of covalent bond between the organophosphorus compound and active site serine of the enzyme [11]. Similarity in thiophosphate moiety led us to evaluate our salicylanilide thiophosphates also against both of these enzymes. This assumption was supported by the fact that salicylanilide carbamates were reported as cholinesterases inhibitors [12].

Based on presented facts, we evaluated salicylanilide diethyl thiophosphates as potential antimycobacterial drugs (salicylanilide derivatives) and anticholinesterase agents (dialkyl aryl phosphorothioate moiety).

## 2. Results and Discussion

### 2.1. Chemistry

**Scheme 1:**
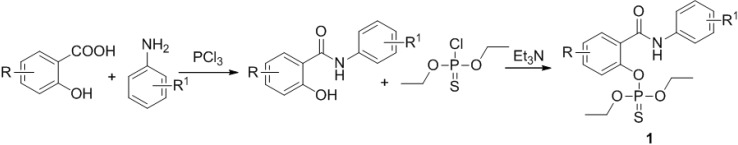
Synthesis of diethyl (2-phenylcarbamoyl)phenyl thiophosphates **1** (R for esters **1** = 4-Cl, 4-Br, 5-Cl; R^1^ = 3-Cl, 4-Cl, 3,4-diCl, 3-Br, 4-Br, 3-F, 4-F, 3-CF_3_, 4-CF_3_.

The synthetic approach to the preparation of the diethyl (2-phenylcarbamoyl)phenyl thiophosphates (phosphorothioates) **1** is depicted in Scheme 1. It is similar to the one used earlier for the synthesis of salicylanilide diethyl phosphates [2]. Starting salicylanilides were routinely obtained by the reaction of 4/5-chloro and 5-bromosalicylic acids with the appropriate anilines mediated by PCl_3_ in chlorobenzene using a microwave reactor [13]. Salicylanilides were esterified by *O*,*O*-diethyl phosphoro-chloridothioate in the presence of triethylamine in dichloromethane (DCM). The synthesis, subsequent isolation and purification gave yields within the 38%–93% range.

### 2.2. In Vitro Antimycobacterial Activity

Antimycobacterial activity was tested *in vitro* against *Mycobacterium tuberculosis* strain 331/88 (H_37_Rv), *Mycobacterium avium* 330/88, *Mycobacterium kansasii* 235/80 and *Mycobacterium kansasii* 6509/96, a strain isolated from a patient. The minimum inhibitory concentration values are presented in the Table 1.

According to the substitution in the salicylic part of the molecule, the tested compounds could be divided into three groups: Group 1 includes 4-Br derivatives **1a–i**; Group 2 includes 4-Cl derivatives **1j**–**r**; and Group 3 contains 5-Cl derivatives **1s–1zz**. It was observed, that halogenation of the position 4 of salicylic part positively contributes to the antimycobacterial activity. This finding correlates with our previous results [2].

**Table 1:** Antimycobacterial activity of salicylanilide diethyl thiophosphates **1**. 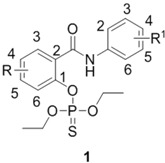

				MIC [µM]									
code	R	R^1^	C log*P*	*Mtb* 331/88 (H_37_Rv)		*M. avium* 330/88		*M. kansasii* 235/80			*M. kansasii* 6509/96		
				14 d	21 d	14 d	21 d	7 d	14 d	21 d	7 d	14 d	21 d
**1a**	4-Br	3-Cl	5.73	**8**	16	500	>500	125	500	500	125	500	500
**1b**	4-Br	4-Cl	5.73	16	16	500	500	125	500	500	125	500	500
**1c**	4-Br	3,4-di-Cl	6.25	**4**	**4**	500	500	62.5	250	500	62.5	125	125
**1d**	4-Br	3-Br	6.00	16	16	125	125	125	125	125	125	125	125
**1e**	4-Br	4-Br	6.00	**8**	16	250	250	125	250	250	62.5	250	250
**1f**	4-Br	3-F	5.35	**8**	16	250	500	62.5	250	500	62.5	250	250
**1g**	4-Br	4-F	5.35	32	32	500	>500	125	500	>500	125	250	250
**1h**	4-Br	3-CF_3_	6.09	**8**	**8**	125	125	32	62.5	125	32	62.5	125
**1i**	4-Br	4-CF_3_	6.09	**8**	**8**	125	125	32	62.5	125	32	62.5	125
**1j**	4-Cl	3-Cl	5.45	32	62.5	>500	>500	250	250	500	250	500	500
**1k**	4-Cl	4-Cl	5.45	32	32	500	500	32	62.5	62.5	62.5	125	125
**1l**	4-Cl	3,4-di-Cl	5.97	**8**	**8**	>500	>500	125	250	500	125	250	250
**1m**	4-Cl	3-Br	5.73	16	16	500	500	125	500	>500	125	500	500
**1n**	4-Cl	4-Br	5.73	32	32	500	500	250	500	500	250	500	500
**1o**	4-Cl	3-F	5.07	32	32	500	500	250	500	>500	250	500	500
**1p**	4-Cl	4-F	5.07	16	16	500	500	250	500	>500	125	250	500
**1q**	4-Cl	3-CF_3_	5.82	**8**	**8**	500	>500	125	500	>500	125	500	500
**1r**	4-Cl	4-CF_3_	5.82	**8**	**8**	500	500	62.5	125	250	62.5	125	250
**1s**	5-Cl	3-Cl	5.45	16	32	125	125	32	125	125	32	62.5	125
**1t**	5-Cl	4-Cl	5.45	32	32	125	125	32	32	32	32	62.5	62.5
**1u**	5-Cl	3,4-di-Cl	5.97	16	16	>500	>500	32	32	32	32	62.5	62.5
**1v**	5-Cl	3-Br	5.73	32	32	500	500	32	125	250	32	125	250
**1w**	5-Cl	4-Br	5.73	32	32	250	250	32	32	32	32	62.5	62.5
**1x**	5-Cl	3-F	5.07	16	32	250	250	32	125	250	32	125	250
**1y**	5-Cl	4-F	5.07	32	32	125	250	62.5	250	500	62.5	250	500
**1z**	5-Cl	3-CF_3_	5.85	32	62.5	500	500	32	125	250	62.5	125	125
**1zz**	5-Cl	4-CF_3_	5.82	**8**	16	**62.5**	**62.5**	**16**	**16**	**16**	**16**	32	32
**INH**				0.5–1	1	>250	>250	>250	>250	>250	2–4	4	4–8
**PAS**				62.5	62.5	32	125	125	1000	>1000	32	125	500

INH = isoniazid; PAS = *para*-aminosalicylic acid. The best MIC values for each strain are given in bold.

Concerning the aniline part of the molecule, the choice of substituents was motivated by our experience with structure-activity relationships observed in the diethyl phosphate series. To summarize the obtained results, there is an obvious contribution of 3- or 4-trifluoromethyl and mainly 3,4-dichloro substitution to the potency against *Mtb*. 3,4-Dichloro derivatives (**1c**, **1l**, **1u**) showed improved potency when compared to either 3-Cl or 4-Cl monosubstituted ones. The most efficient compound was *O*-{4-bromo-2-[(3,4-dichlorophenyl)carbamoyl]phenyl} *O*,*O*-diethyl phosphorothioate **1c** with MIC of 4 µM. Five of the compounds, 3-trifluoromethyl (**1h**, **1q**), 4-trifluoromethyl (**1i**, **1r**) and 3,4-dichloro (**1l**) derivatives also showed significant MIC of 8 µM. Even though the structure-activity relationships are in correlation with salicylanilide diethyl phosphates [2], diethyl thiophosphate compounds expressed only moderate antimycobacterial activity against *Mtb* and atypical mycobacteria. The introduction of sulphur into the phosphate group did not meet our expectations about increased antimycobacterial activity, not only against *Mtb*, but also against *M. avium* and *M. kansasii*, which were inhibited even at higher concentrations (≥ 16 µM) than *Mtb*. The best activity against these last mentioned strains was described for **1zz**. *M. avium* showed the lowest susceptibility to salicylanilide phosphates with MIC values ≥ 62.5 µM.

None of the diethyl thiophosphates **1** exhibited the activity similar to isoniazid (INH) against *M. tuberculosis* and one strain of *M. kansasii* (6509/96). On the other hand, ten salicylanilide derivatives (**1d**, **1e**, **1h**, **1i**, **1s**, **1t**, **1w**–**1y**, and **1zz**) were significantly more active against INH-resistant *M. avium* (with all MIC values ≤ 250 μM) and fourteen molecules (**1d**, **1e**, **1h**, **1i**, **1k**, **1r**–**1x**, **1z**, and **1zz**) share MICs for *M. kansasii* 235/80 lower (≤ 250 μM) than INH. All diethyl thiophosphates **1** outstripped the activity of *para*-aminosalicylic acid (PAS), a second-line oral antimycobacterial drug sharing a structural similarity, against *Mtb* and none of them exhibited markedly lower growth inhibition of *M. kansasii* 235/80. The inverse relationship was discovered for *M. avium*—only *O*-(5-chloro-2-{[4-(trifluoromethyl)phenyl]carbamoyl}phenyl) *O*,*O*-diethyl phosphorothioate **1zz** showed an equal *in vitro* potency.

### 2.3. In Vitro AChE a BChE Inhibition

The ability of the tested compounds to inhibit AChE from electric eel (*Electrophorus electricus* L.) and BChE from equine serum was screened *in vitro* using modified Ellman’s method for the evaluation of cholinesterase activity. The effectiveness of the inhibitors is expressed as IC_50_, representing the concentration of an inhibitor required for 50% inhibition of the enzyme. The activities of the tested compounds were compared with standards rivastigmine and galanthamine (Table 2). These two standards were chosen due to their different structures. Rivastigmine is an acylating pseudo-reversible carbamate inhibitor of cholinesterases that inhibits AChE as well as BChE, while galanthamine is a non-acylating competitive reversible cholinesterase inhibitor. Furthermore, galanthamine acts as an allosteric ligand at nicotinic acetylcholine receptors. The choice of these drugs with different mechanism of action can provide relevant results. All tested compounds expressed a good inhibitory activity with IC_50_s in low micromolar values. These values are summarized in Table 2.

It is possible to conclude that most of the tested compounds inhibit AChE somewhat better than BChE. Derivatives of Group 2 (R = 4-Cl) and Group 3 (R = 5-Cl) are more effective inhibitors of AChE than derivatives of Group 1 (R = 4-Br). For Groups 1 and 3, the relationship is inverse when compared to anti-TB activity.

**Table 2:** The IC_50_ values of thiophosphates **1** for AChE and BChE inhibition.

Code		IC_50_ for AChE [µM]			IC_50_ for BChE [µM]	
**1a**		12.60 ± 0.05			13.90 ± 0.05	
**1b**		12.9 ± 0.1			13.5 ± 0.9	
**1c**		14.0 ± 0.1			18.8 ± 0.7	
**1d**		11.30 ± 0.05			20.0 ± 0.2	
**1e**		10.7 ± 0			18.9 ± 0.2	
**1f**		12.70 ± 0.05			12.2 ± 0.0	
**1g**		12.40 ± 0.05			12.6 ± 0.3	
**1h**		13.0 ± 0.02			20.0 ± 0.5	
**1i**		11.20 ± 0.02			20.2 ± 1.6	
**1j**		9.97 ± 0.24			14.80 ± 0.15	
**1k**		9.78 ± 0.11			12.70 ± 0.10	
**1l**		9.33 ± 0.11			17.4 ± 0.5	
**1m**		14.1 ± 0.2			15.1 ± 1.3	
**1n**		9.32 ± 0.06			15.1 ± 0.1	
**1o**		9.94 ± 0.20			11.8 ± 0.6	
**1p**		13.6 ± 1.1			14.9 ± 0.8	
**1q**		12.1 ± 0.2			19.9 ± 0.2	
**1r**		9.70 ± 0.18			15.6 ± 0.7	
**1s**		9.79 ± 0.20			12.5 ± 0.1	
**1t**		10.8 ± 0.3			13.80 ± 0.05	
**1u**		9.56 ± 0.21			14.7 ± 0.2	
**1v**		9.98 ± 0.43			14.9 ± 1.0	
**1w**		10.12 ± 0.08			16.4 ± 0.2	
**1x**		**8.04 ± 0.27**			**8.68 ± 0.54**	
**1y**		11.6 ± 0.3			**8.67 ± 0.49**	
**1z**		11.4 ± 0.3			15.0 ± 0	
**1zz**		9.16 ± 0.17			18.3 ± 1.0	
**Rivastigmine**		501 ± 3.08			19.95 ± 0.20	
**Galanthamine**		4 ± 0.13			7.96 ± 0.59	

AChE and BChE inhibition is expressed as mean ± SD (n = 3 experiments). The best values for each enzyme are given in bold. The values of IC_50_ of rivastigmine and galanthamine were published previously [14].

As in the case of the inhibition of BChE, there is no observed effect of aniline substitution by chlorine or bromine on the power of inhibition. Both 3- and 4-fluorine represent the most convenient substitution patterns for the aniline ring for BChE (**1f**, **1g**, **1o**, **1p**, and especially **1x** and **1y**). However, IC_50_s of all derivatives are within a narrow concentration range (8–20 µM) without any sharp differences. We presume that this is a consequence of their mechanism of action—irreversible phosphorylation of enzyme active site; from this point of view, salicylanilide core serves as a carrier of phosphorylating agents. In general, the IC_50_ values of all diethyl thiophosphates **1** were within a narrow concentration range without any sharp differences: 8.04–14.1 μM for AChE and 8.67–20.2 μM for BChE. *O*-{5-Chloro-2-[(3-fluorophenyl)carbamoyl]phenyl} *O*,*O*-diethyl phosphorothioate (**1x**) was the most effective AChE inhibitor (IC_50_ = 8.04 ± 0.27 μM), and the second best one for inhibition of butyrylcholinesterase (IC_50_ = 8.68 ± 0.54 μM). A bit more effective BChE inhibitor is *O*-{5-chloro-2-[(4-fluorophenyl)-carbamoyl]phenyl} *O*,*O*-diethyl phosphorothioate (**1y**) (IC_50_ = 8.67 ± 0.49 μM). Then again the least effective AChE inhibitor is *O*-{2-[(3-bromophenyl)carbamoyl]-4-chlorophenyl} *O*,*O*-diethyl phosphorothioate (**1m**, IC_50_ = 14.1 ± 0.20 μM) and the least effective BChE inhibitor was *O*-(4-bromo-2-{[4-(trifluoromethyl)phenyl]carbamoyl}phenyl) *O*,*O*-diethyl phosphorothioate (**1i**, IC_50_ = 20.2 ± 1.6 µM). Salicylanilide diethyl thiophosphates provide significantly lower IC_50_ values for AChE than salicylanilide *N*-alkyl carbamates [12].

## 3. Experimental Section

### 3.1. General Information

Solvents and reagents were purchased from Sigma-Aldrich (Darmstadt, Germany) and Penta Chemicals (Prague, Czech Republic) and were used as received. Reactions were monitored by thin-layer chromatography (TLC) using commercially available coated plates (Merck Kieselgel 60 F254 silica) with UV light (256 nm) visualization. The melting points were determined on a Büchi Melting Point B-540 apparatus (Bűchi Labortechnik AG, Flawil, Switzerland) using open capillaries and were uncorrected. IR spectra were recorded on a Nicolet 6700 FT-IR spectrometer (Thermo Fisher Scientific, Waltham, MA, USA) in the range of 400–4000 cm^−1^ using the ATR technique. The NMR spectra were recorded at ambient temperature on a Varian Mercury (500 MHz for ^1^H, 125 MHz for ^13^C and 202 MHz for ^31^P; Varian Comp. Palo Alto, CA, USA) using deuterated chloroform (CDCl_3_) solutions of the samples. Proton spectra are referenced to TMS as the internal standard; the carbon shifts are given against the central line of the solvent signal (CDCl_3_ at *δ* = 77.1 ppm). The coupling constants (*J*) are reported in Hz. Elementary analysis was performed on CE Instruments EA-1110 CHN analyser (CE Instruments, Wigan, UK). The calculated log*P* values (Clog*P*), which are the logarithms of the partition coefficients for octan-1-ol/water, were determined using the program CS ChemOffice Ultra version 12.0 (CambridgeSoft, Cambridge, MA, USA).

### 3.2. Chemistry

*General Procedure for the Synthesis of Diethyl (2-phenylcarbamoyl)thiophosphates*

Appropriate salicylanilide (2.0 mmol) was suspended at 20 °C in dichloromethane (10 mL) and then 1.5 equivalents of triethylamine (0.418 mL; 3.0 mmol) were added under vigorous stirring. After 5 min, 1.2 equivalents of *O*,*O*-diethyl phosphorochloridothioate (0.347 mL) was added to the mixture. The mixture was stirred at the room temperature for 2 h; the reaction was monitored by TLC using a 4:1 toluene/ethyl acetate mixture as eluent. After this time, the solution was added to a chromatography column using chloroform or chloroform/hexane 9:1 as eluent. The solvent was removed under reduced pressure to obtain the product, which was recrystallized from acetone-hexane, if necessary.

*O-{4-Bromo-2-[(3-chlorophenyl)carbamoyl]phenyl} O,O-diethyl phosphorothioate* (**1a**). Yield: 86%, white solid; m.p. 55–56 °C; IR: 1678 (amide I), 1593 (ν CC_aromatic_), 1529 (amide II), 1478 (ν CC_aromatic_) cm^−1^; ^1^H-NMR: δ 9.06 (1H, bs, NH), 8.15 (1H, dd, *J* = 2.5 Hz, *J* = 1.0 Hz, H3), 7.86 (1H, t, *J* = 2.0 Hz, H2'), 7.61–7.57 (2H, m, H5, H6'), 7.30–7.26 (2H, m, H6, H5'), 7.14–7.11 (1H, m, H4'), 4.28–4.16 (4H, m, CH_A_H_B_), 1.32 (6H, dt, *J* = 7.0 Hz, 0.9 Hz, CH_3_); ^13^C-NMR: δ 161.4, 146.7 (1C, d, *J* = 8.1 Hz), 139.0, 135.4 (1C, d, *J* = 1.5 Hz), 134.6, 134.5, 130.0, 128.5 (1C, d, *J* = 6.1 Hz), 124.6, 122.9 (1C, d, *J* = 3.0 Hz), 120.1, 119.1 (1C, d, *J* = 2.0 Hz), 118.0, 66.0 (2C, d, *J* = 5.6 Hz, CH_A_H_B_), 15.8 (2C, d, *J* = 7.4 Hz, CH_3_); ^31^P-NMR: δ 62.6. Anal. Calcd. for C_17_H_18_BrClNO_4_PS (478.72): C, 42.65; H, 3.79; N, 2.93. Found: C, 42.45; H, 4.00; N, 2.96.

*O-{4-Bromo-2-[(4-chlorophenyl)carbamoyl]phenyl} O,O-diethyl phosphorothioate* (**1b**). Yield: 89%, white solid; m.p. 75–77 °C; IR: 1666 (amide I), 1592 (ν CC_aromatic_), 1530 (amide II), 1492, 1478 (ν CC_aromatic_) cm^−1^; ^1^H-NMR: δ 9.05 (1H, bs, NH), 8.14 (1H, dd, *J* = 2.5 Hz, 1.0 Hz, H3), 7.72–7.69 (2H, m, H2', H6'), 7.58 (1H, dd, *J* = 8.5 Hz, 2.5 Hz, H5), 7.34–7.31 (2H, m, H3', H5'), 7.27 (1H, dd, *J* = 8.5 Hz, 1.0 Hz, H6), 4.28–4.15 (4H, m, CH_A_H_B_), 1.31 (6H, dt, *J* = 7.0 Hz, 0.7 Hz, CH_3_); ^13^C-NMR: δ 161.4, 146.6 (1C, d, *J* = 8.5 Hz), 136.4, 135.3 (1C, d, *J* = 1.4 Hz), 134.5, 129.6, 129.0, 128.7 (1C, d, *J* = 6.3 Hz), 122.9 (1C, d, *J* = 2.8 Hz), 121.3, 119.0 (1C, d, *J* = 2.0 Hz), 66.0 (2C, d, *J* = 5.5 Hz, CH_A_H_B_), 15.8 (2C, d, *J* = 7.4 Hz, CH_3_); ^31^P-NMR: δ 62.6. Anal. Calcd for C_17_H_18_BrClNO_4_PS (478.72): C, 42.65; H, 3.79; N, 2.93. Found: C, 42.76; H, 3.70; N, 2.99.

*O-{4-Bromo-2-[(3,4-dichlorophenyl)carbamoyl]phenyl} O,O-diethyl phosphorothioate* (**1c**). Yield: 69%, white solid; m.p. 80–81 °C; IR: 1678 (amide I), 1585 (ν CC_aromatic_), 1525 (amide II), 1476 (ν CC_aromatic_) cm^−1^; ^1^H-NMR: δ 9.13 (1H, bs, NH), 8.15 (1H, dd, *J* = 2.5 Hz, 1.0 Hz, H3), 8.00 (1H, d, *J* = 2.5 Hz, H2'), 7.61–7.57 (2H, m, H5, H6'), 7.41 (1H, d, *J* = 8.5 Hz, H5'), 7.28 (1H, dd, *J* = 8.5 Hz, 1.0 Hz, H6), 4.29–4.16 (4H, m, CH_A_H_B_), 1.32 (6H, dt, *J* = 7.0 Hz, 1.0 Hz, CH_3_); ^13^C-NMR: δ 161.4, 146.7 (1C, d, *J* = 8.4 Hz), 137.3, 135.6 (1C, d, *J* = 1.5 Hz), 134.6, 132.8, 130.5, 128.1 (1C, d, *J* = 6.1 Hz), 127.8, 122.9 (1C, d, *J* = 2.9 Hz), 121.7, 119.3, 119.1 (1C, d, *J* = 1.9 Hz), 66.1 (2C, d, *J* = 5.5 Hz, CH_A_H_B_), 15.8 (2C, d, *J* = 7.3 Hz, CH_3_); ^31^P-NMR: δ 62.5. Anal. Calcd for C_17_H_17_BrCl_2_NO_4_PS (513.17): C, 39.79; H, 3.34; N, 2.73. Found: C, 39.88; H, 3.46; N, 2.85.

*O-{4-Bromo-2-[(3-bromophenyl)carbamoyl]phenyl} diethyl phosphate* (**1d**). Yield: 63%, white solid; m.p. 67–68 °C (acetone-hexane); IR: 1677 (amide I), 1591 (ν CC_aromatic_), 1530 (amide II), 1476 (ν CC_aromatic_) cm^−1^; ^1^H-NMR: δ 9.05 (1H, bs, NH), 8.15 (1H, dd, *J* = 2.5 Hz, 1.0 Hz, H3), 8.00 (1H, t, *J* = 1.8 Hz, H2'), 7.67–7.64 (1H, m, H6'), 7.59 (1H, dd, *J* = 8.5 Hz, 2.5 Hz, H5), 7.30–7.21 (3H, m, H6, H4', H5'), 4.29–4.16 (4H, m, CH_A_H_B_), 1.32 (6H, dt, *J* = 7.0 Hz, 1.0 Hz, CH_3_); ^13^C-NMR: δ 161.4, 146.7 (1C, d, *J* = 8.3 Hz), 139.1, 135.4 (1C, d, *J* = 1.5 Hz), 134.6, 130.3, 128.5 (1C, d, *J* = 6.3 Hz), 127.6, 122.9, 122.9 (1C, d, *J* = 2.9 Hz), 122.6, 119.1 (1C, d, *J* = 1.9 Hz), 118.5, 66.0 (2C, d, *J* = 5.5 Hz, CH_A_H_B_), 15.8 (2C, d, *J* = 7.4 Hz, CH_3_); ^31^P-NMR: δ 62.6. Anal. Calcd for C_17_H_18_Br_2_NO_4_PS (523.18): C, 39.03; H, 3.47; N, 2.68. Found: C, 39.14; H, 3.34; N, 2.55.

*O-{4-Bromo-2-[(4-bromophenyl)carbamoyl]phenyl} O,O-diethyl phosphorothioate* (**1e**). Yield: 69%, white solid; m.p. 82–83 °C (acetone-hexane); IR: 1666 (amide I), 1589 (ν CC_aromatic_), 1527 (amide II), 1488, 1441 (ν CC_aromatic_) cm^−1^; ^1^H-NMR: δ 9.05 (1H, bs, NH), 8.14 (1H, dd, *J* = 2.5 Hz, 1.0 Hz, H3), 7.67–7.64 (2H, m, H2', H6'), 7.58 (1H, dd, *J* = 8.5 Hz, *J* = 2.5 Hz, H5), 7.49–7.45 (2H, m, H3', H5'), 7.27 (1H, dd, *J* = 8.5 Hz, 1.3 Hz, H6), 4.27–4.15 (4H, m, CH_A_H_B_), 1.31 (6H, dt, *J* = 7.0 Hz, 1.0 Hz, CH_3_); ^13^C-NMR: δ 161.4, 146.6 (1C, d, *J* = 8.4 Hz), 136.9, 135.3 (1C, d, *J* = 1.4 Hz), 134.5, 132.0, 128.7 (1C, d, *J* = 6.0 Hz), 122.9 (1C, d, *J* = 2.9 Hz), 121.6, 119.1 (1C, d, *J* = 1.9 Hz), 117.2, 66.0 (2C, d, *J* = 5.5 Hz, CH_A_H_B_), 15.8 (2C, d, *J* = 7.4 Hz, CH_3_); ^31^P-NMR: δ 62.5. Anal. Calcd for C_17_H_18_Br_2_NO_4_PS (523.18): C, 39.03; H, 3.47; N, 2.68. Found: C, 39.18; H, 3.34; N, 2.71.

*O-{4-Bromo-2-[(3-fluorophenyl)carbamoyl]phenyl} O,O-diethyl phosphorothioate* (**1f**). Yield: 73%, oily liquid; IR: 1680 (amide I), 1606 (ν CC_aromatic_), 1540 (amide II), 1492, 1473 (ν CC_aromatic_) cm^−1^; ^1^H-NMR: δ 9.09 (1H, bs, NH), 8.15 (1H, d, *J* = 2.5 Hz, H3), 7.70 (1H, d, *J* = 11.0 Hz, H6'), 7.58 (1H, dd, *J* = 8.5 Hz, 2.5 Hz, H5), 7.41–7.25 (2H, m, H6, H2', H5'), 6.85 (1H, td, *J* = 8.5 Hz, 1.8 Hz, H4'), 4.26–4.17 (4H, m, CH_A_H_B_), 1.31 (6H, t, *J* = 7.0 Hz, CH_3_); ^13^C-NMR: δ 162.9 (1C, d, *J* = 243.3 Hz, C3'), 161.5, 146.6 (1C, d, *J* = 8.4 Hz), 139.3 (1C, d, *J* = 10.8 Hz), 135.3 (1C, d, *J* = 1.5 Hz), 134.5, 130.0 (1C, d, *J* = 9.3 Hz), 128.6 (1C, d, *J* = 6.1 Hz), 122.9 (1C, d, *J* = 3.0 Hz), 119.0 (1C, d, *J* = 2.0 Hz), 115.3 (1C, d, *J* = 2.9 Hz), 111.3 (1C, d, *J* = 21.4 Hz), 107.5 (1C, d, *J* = 26.5 Hz), 66.0 (2C, d, *J* = 5.5 Hz, CH_A_H_B_), 15.8 (2C, d, *J* = 7.4 Hz, CH_3_); ^31^P-NMR: δ 62.6. Anal. Calcd for C_17_H_18_BrFNO_4_PS (462.27): C, 44.17; H, 3.92; N, 3.03. Found: C, 44.03; H, 4.05; N, 3.20.

*O-{4-Bromo-2-[(4-fluorophenyl)carbamoyl]phenyl} O,O-diethyl phosphorothioate* (**1g**). Yield: 49%, oily liquid; IR: 1664 (amide I), 1614 (ν CC_aromatic_), 1541 (amide II), 1509, 1470 (ν CC_aromatic_) cm^−1^; ^1^H-NMR: δ 9.00 (1H, bs, NH), 8.14 (1H, dd, *J* = 2.5 Hz, 1.0 Hz, H3), 7.73–7.69 (2H, m, H2', H6'), 7.57 (1H, dd, *J* = 8.7 Hz, 2.5 Hz, H5), 7.28 (1H, dd, *J* = 8.5 Hz, 1.0 Hz, H6), 7.08–7.03 (2H, m, H3', H5'), 4.27–4.16 (4H, m, CH_A_H_B_), 1.30 (6H, dt, *J* = 7.0 Hz, 1.0 Hz, CH_3_); ^13^C-NMR: δ 161.3, 159.5 (1C, d, *J* 242.6 Hz, C4'), 146.6 (1C, d, *J* = 8.5 Hz), 135.2 (1C, d, *J* = 1.5 Hz), 134.5, 133.9 (1C, d, *J* = 2.9 Hz, C1'), 128.80 (1C, d, *J* = 6.1 Hz), 122.9 (1C, d, *J* = 3.0 Hz), 121.7 (2C, d, *J* = 7.8 Hz, C2', C6'), 119.0 (1C, d, *J* = 2.0 Hz), 115.6 (2C, d, *J* = 22.4 Hz, C3', C5'), 65.9 ( 2C, d, *J* = 5.6 Hz, CH_A_H_B_), 15.8 (2C, d, *J* = 7.4 Hz, CH_3_); ^31^P-NMR: δ 62.6. Anal. Calcd for C_17_H_18_BrFNO_4_PS (462.27): C, 44.17; H, 3.92; N, 3.03. Found: C, 44.30; H, 3.99; N, 2.87.

*O-(4-Bromo-2-{[3-(trifluoromethyl)phenyl]carbamoyl}phenyl) O,O-diethyl phosphorothioate* (**1h**). Yield: 38%, oily liquid; IR: 1677 (amide I), 1606 (ν CC_aromatic_), 1537 (amide II), 1492, 1472 (ν CC_aromatic_) cm^−1^; ^1^H-NMR: δ 9.21 (1H, bs, NH), 8.19 (1H, dd, *J* = 2.8 Hz, 1.1 Hz, H3), 8.04 (1H, s, H2'), 7.97 (1H, d, *J* = 8.2 Hz, H6'), 7.60 (1H, dd, *J* = 8.7 Hz, 2.6 Hz, H5), 7.49 (1H, t, *J* = 8.0 Hz, H5'), 7.41 (1H, d, *J* = 7.8 Hz, H4'),7.31 (1H, dd, *J* = 8.7 Hz, 1.3 Hz, H6), 4.29–4.17 (4H, m, CH_A_H_B_), 1.31 (6H, t, *J* = 7.1 Hz, CH_3_); ^13^C-NMR: δ 161.55, 146.76 (1C, d, *J* = 8.2 Hz), 138.39, 135.51 (1C, d, *J* = 1.8 Hz), 134.64 (1C, d, *J* = 1.0 Hz), 131.39 (1C, q, *J* = 32.5 Hz, C3'), 129.57, 128.27 (1C, d, *J* = 6.2 Hz), 123.83 (1C, q, *J* = 272.5 Hz, CF_3_), 123.12, 122.89 (1C, d, *J* = 2.9 Hz), 121.12 (1C, q, *J* = 3.9 Hz, C2'), 119.09 (1C, d, *J* = 2.1 Hz), 116.78 (1C, q, *J* = 3.9 Hz, C4'), 66.05 (2C, d, *J* = 5.6 Hz, CH_A_H_B_), 15.76 (2C, d, *J* = 7.3 Hz, CH_3_); ^31^P-NMR: δ 62.7. Anal. Calcd for C_18_H_18_BrF_3_NO_4_PS (512.28): C, 42.20; H, 3.54; N, 2.73. Found: C, 42.41; H, 3.59; N, 2.64. 

*O-(4-Bromo-2-{[4-(trifluoromethyl)phenyl]carbamoyl}phenyl) O,O-diethyl phosphorothioate* (**1i**). Yield: 61%, white solid; m.p. 83–84 °C (acetone-hexane); IR: 1683 (amide I), 1604 (ν CC_aromatic_), 1540 (amide II), 1472 (ν CC_aromatic_) cm^−1^; ^1^H-NMR: δ 9.23 (1H, bs, NH), 8.17 (1H, dd, *J* = 2.5 Hz, 1.3 Hz, H3), 7.89 (2H, d, *J* = 8.5 Hz, H3', H5'), 7.62 (2H, d, *J* = 8.5 Hz, H2', H6'), 7.60 (1H, dd, *J* = 9.0 Hz, 2.5 Hz, H5), 7.23 (1H, dd, *J* = 9.0 Hz, 1.3 Hz, H6), 4.29–4.16 (4H, m, CH_A_H_B_), 1.31 (6H, dt, *J* = 7.0 Hz, 1.0 Hz, CH_3_); ^13^C-NMR: δ 161.6, 146.7 (1C, d, *J* = 8.5 Hz), 140.9 (1C, d, *J* = 1.1 Hz), 135.5 (1C, d, *J* = 1.6 Hz), 134.6, 128.4 (1C, d, *J* = 6.1 Hz), 126.3 (2C, q, *J* = 3.8 Hz, C3', C5'), 126.3 (1C, q, *J* = 32.8 Hz, C4'), 124.0 (1C, q, *J* = 270.1 Hz, CF_3_), 122.9 (1C, d, *J* = 2.9 Hz), 119.7, 119.1 (1C, d, *J* = 2.0 Hz), 66.0 (2C, d, *J* =5.6 Hz, CH_A_H_B_), 15.8 (2C, d, *J* = 7.4 Hz, CH_3_); ^31^P-NMR: δ 62.5. Anal. Calcd for C_18_H_18_BrF_3_NO_4_PS (512.28): C, 42.20; H, 3.54; N, 2.73. Found: C, 42.39; H, 3.38; N, 2.91.

*O-{4-Chloro-2-[(3-chlorophenyl)carbamoyl]phenyl} O,O-diethyl phosphorothioate* (**1j**). Yield: 75%, white solid; m.p. 52–54 °C; IR: 1677, 1658 (amide I), 1597 (ν CC_aromatic_), 1540 (amide II), 1476, 1428 (ν CC_aromatic_) cm^−1^; ^1^H-NMR: δ 9.07 (1H, bs, NH), 8.00 (1H, dd, *J* = 2.8 Hz, 1.3 Hz, H3), 7.87 (1H, t, *J* = 2.0 Hz, H2'), 7.59 (1H, dd, *J* = 8.3 Hz, 1.3 Hz, H6'), 7.43 (1H, dd, *J* = 8.8 Hz, 2.8 Hz, H5), 7.35 (1H, dd, *J* = 8.5 Hz, 1.5 Hz, H6), 7.28 (1H, t, *J* = 8.0 Hz, H5'), 7.14–7.11 (1H, m, H4'), 4.28–4.16 (4H, m, CH_A_H_B_), 1.32 (6H, dt, *J* = 7.0 Hz, 0.9 Hz, CH_3_); ^13^C-NMR: δ 161.5, 146.1 (1C, d, *J* = 8.4 Hz), 139.0, 134.6, 132.4 (1C, d, *J* = 1.8 Hz), 131.6, 131.6 (1C, d, *J* = 1.9 Hz), 130.0, 128.2 (1C, d, *J* = 6.1 Hz), 124.6, 122.6 (1C, d, *J* = 3.0 Hz), 120.1, 118.0, 66.0 (2C, d, *J* = 5.6 Hz, CH_A_H_B_), 15.8 (2C, d, *J* = 7.5 Hz, CH_3_); ^31^P-NMR: δ 62.7. Anal. Calcd for C_17_H_18_Cl_2_NO_4_PS (434.27): C, 47.02; H, 4.18; N, 3.23. Found: C, 47.15; H, 4.08; N, 3.32.

*O-{4-Chloro-2-[(4-chlorophenyl)carbamoyl]phenyl} O,O-diethyl phosphorothioate* (**1k**). Yield: 62%, white solid; m.p. 53–54 °C; IR: 1666 (amide I), 1594 (ν CC_aromatic_), 1536 (amide II), 1492 (ν CC_aromatic_) cm^−1^; ^1^H-NMR: δ 9.06 (1H, bs, NH), 7.99 (1H, d, *J* = 2.5 Hz, H3), 7.71 (2H, d, *J* = 8.5 Hz, H2', H6'), 7.43 (1H, dd, *J* = 8.8 Hz, 2.5 Hz, H5), 7.35–7.29 (3H, m, H6, H3', H5'), 4.28–4.15 (4H, m, CH_A_H_B_), 1.30 (6H, t, *J* = 7.0 Hz, CH_3_); ^13^C-NMR: δ 161.5, 146.1 (1C, d, *J* = 8.6 Hz), 136.4, 132.3, 131.5, 129.5, 129.0, 128.4 (1C, d, *J* = 6.3 Hz), 122.6 (1C, d, *J* = 2.6 Hz), 121.3, 66.0 (2C, d, *J* = 5.5 Hz, CH_A_H_B_), 15.8 (2C, d, *J* = 7.4 Hz, CH_3_); ^31^P-NMR: δ 62.6. Anal. Calcd for C_17_H_18_Cl_2_NO_4_PS (434.27): C, 47.02; H, 4.18; N, 3.23. Found: C, 47.12; H, 4.30; N, 3.17. 

*O-{4-Chloro-2-[(3,4-dichlorophenyl)carbamoyl]phenyl} O,O-diethyl phosphorothioate* (**1l**). Yield: 87%, white solid; m.p. 90–91 °C (acetone-hexane); IR: 1677 (amide I), 1586 (ν CC_aromatic_), 1524 (amide II), 1477 (ν CC_aromatic_) cm^−1^; ^1^H-NMR: δ 9.14 (1H, bs, NH), 8.01–7.99 (2H, m, H3, H2'), 7.59 (1H, dd, *J* = 9.0 Hz, 2.5 Hz, H6'), 7.44 (1H, dd, *J* = 8.8 Hz, 2.8 Hz, H5), 7.41 (1H, dd, *J* = 9.0 Hz, 0.8 Hz, H5'), 7.34 (1H, dd, *J* = 8.8 Hz, 1.0 Hz, H6), 4.29–4.17 (4H, m, CH_A_H_B_), 1.32 (6H, tt, *J* = 7.2 Hz, 0.9 Hz, CH_3_); ^13^C-NMR: δ 161.5, 146.1 (1C, d, *J* = 8.5 Hz), 137.3, 132.8, 132.6 (1C, d, *J* = 1.8 Hz), 131.7, 131.6 (1C, d, *J* = 2.0 Hz), 130.5, 127.9 (1C, d, *J* = 6.3 Hz), 127. 8, 122.6 (1C, d, *J* = 3.0 Hz), 121.7, 119.3, 66.1 (2C, d, *J* = 5.5 Hz, CH_A_H_B_), 15.8 (2C, d, *J* = 7.4 Hz, CH_3_); ^31^P-NMR: δ 62.6. Anal. Calcd for C_17_H_17_Cl_3_NO_4_PS (468.72): C, 43.56; H, 3.66; N, 2.99. Found: C, 43.44; H, 3.57; N, 3.16.

*O-{2-[(3-Bromophenyl)carbamoyl]-4-chlorophenyl} O,O-diethyl phosphorothioate* (**1m**). Yield: 76%, white solid; m.p. 57–58 °C (acetone-hexane); IR: 1677 (amide I), 1592 (ν CC_aromatic_), 1530 (amide II), 1476 (ν CC_aromatic_) cm^−1^; ^1^H-NMR: δ 9.06 (1H, bs, NH), 8.01–7.99 (2H, m, H3, H2'), 7.66 (1H, ddd, *J* = 8.0 Hz, 2.0 Hz, 1.0 Hz, H6'), 7.44 (1H, dd, *J* = 8.8 Hz, 2.8 Hz, H5), 7.35 (1H, dd, *J* = 8.8 Hz, 1.3 Hz, H6), 7.28 (1H, ddd, *J* = 8.0 Hz, 2.0 Hz, 1.0 Hz, H4'), 7.22 (1H, t, *J* = 8.0 Hz, H5'), 4.28–4.16 (4H, m, CH_A_H_B_), 1.32 (6H, dt, *J* = 7.0 Hz, 1.0 Hz, CH_3_); ^13^C-NMR: δ 161.5, 146.1 (1C, d, *J* = 8.3 Hz), 139.1, 132.4 (1C, d, *J* = 1.5 Hz), 131.6, 131.6 (1C, d, *J* = 1.8 Hz), 130.3, 128.2 (1C, d, *J* = 6.1 Hz), 127.6, 122.9, 122.6 (2C, d, *J* = 3.0 Hz), 122.6, 118.5, 66.0 (2C, d, *J* = 5.5 Hz, CH_A_H_B_), 15.8 (2C, d, *J* = 7.4 Hz, CH_3_); ^31^P-NMR: δ 62.7. Anal. Calcd for C_17_H_18_BrClNO_4_PS (478.72): C, 42.65; H, 3.79; N, 2.93. Found: C, 42.79; H, 3.65; N, 2.82.

*O-{2-[(4-Bromophenyl)carbamoyl]-4-chlorophenyl} O,O-diethyl phosphorothioate* (**1n**). Yield: 81%, white solid; m.p. 60–62 °C; IR: 1677, 1660 (amide I), 1600, 1572 (ν CC_aromatic_), 1538 (amide II), 1489 (ν CC_aromatic_) cm^−1^; ^1^H-NMR: δ 9.06 (1H, bs, NH), 7.99 (1H, dd, *J* = 2.7 Hz, 1.2 Hz, H3), 7.68–7.63 (2H, m, H2', H6'), 7.49–7.45 (2H, m, H3', H5'), 7.43 (1H, dd, *J* = 8.8 Hz, 2.7 Hz, H5), 7.33 (1H, dd, *J* = 8.8 Hz, 1.3 Hz, H6), 4.27–4.15 (4H, m, CH_A_H_B_), 1.31 (6H, dt, *J* = 7.0 Hz, 0.8 Hz, CH_3_); ^13^C-NMR: δ 161.8, 146.1 (1C, d, *J* = 8.4 Hz), 137.0, 132.3 (1C, d, *J* = 1.8 Hz), 132.0, 131.6 (1C, d, *J* = 2.0 Hz), 128.4 (1C, d, *J* = 6.3 Hz), 122.6 (1C, d, *J* = 2.9 Hz), 121.6, 117.2, 66.0 (2C, d, *J* = 5.5 Hz, CH_A_H_B_), 15.8 (2C, d, *J* = 7.5 Hz, CH_3_); ^31^P-NMR: δ 62.7. Anal. Calcd for C_17_H_18_BrClNO_4_PS (478.72): C, 42.65; H, 3.79; N, 2.93. Found: C, 42.49; H, 4.01; N, 3.10.

*O-{4-Chloro-2-[(3-fluorophenyl)carbamoyl]phenyl} O,O-diethyl phosphorothioate* (**1o**). Yield: 65%, white solid; m.p. 46–48 °C; IR: 1659 (amide I), 1612 (ν CC_aromatic_), 1548 (amide II), 1473, 1446 (ν CC_aromatic_) cm^−1^; ^1^H-NMR: δ 9.10 (1H, bs, NH), 8.00 (1H, dd, *J* = 2.5 Hz, 1.0 Hz, H3), 7.70 (1H, dt, *J* = 11.0 Hz, 2.2 Hz, H6'), 7.43 (1H, dd, *J* = 8.8 Hz, 2.5 Hz, H5), 7.35 (1H, dd, *J* = 8.8 Hz, 1.0 Hz, H6), 7.36–7.27 (2H, m, H2', H5'), 6.85 (1H, ddt, *J* = 8.0 Hz, 2.5 Hz, *J* = 0.9 Hz, H4'), 4.28–4.16 (4H, m, CH_A_H_B_), 1.31 (6H, dt, *J* = 7.0 Hz, 1.0 Hz, CH_3_); ^13^C-NMR: δ 162.9 (1C, d, *J* = 243.5 Hz, C3'), 161.6, 146.1 (1C, d, *J* = 8.4 Hz), 139.3 (1C, d, *J* = 10.9 Hz), 132.3 (1C, d, *J* = 1.6 Hz), 131.6 (1C, d, *J* = 1.3 Hz), 131.5, 130.0 (1C, d, *J* = 9.3 Hz), 128.3 (1C, d, *J* = 6.1 Hz), 122.6 (1C, d, *J* = 2.9 Hz), 115.3 (1C, d, *J* = 3.0 Hz, C6'), 111.3 (1C, d, *J* = 21.3 Hz), 107.5 (1C, d, *J* = 26.4 Hz), 66.0 (2C, d, *J* = 5.5 Hz, CH_A_H_B_), 15.8 (2C, d, *J* = 7.4 Hz, CH_3_); ^31^P-NMR: δ 62.7. Anal. Calcd for C_17_H_18_ClFNO_4_PS (417.82): C, 48.87; H, 4.34; N, 3.35. Found: C, 48.75; H, 4.23; N, 3.52.

*O-{4-Chloro-2-[(4-fluorophenyl)carbamoyl]phenyl} O,O-diethyl phosphorothioate* (**1p**). Yield: 68%, liquid; IR: 1670 (amide I), 1615 (ν CC_aromatic_), 1541 (amide II), 1509, 1473 (ν CC_aromatic_) cm^−1^; ^1^H-NMR: δ 9.02 (1H, bs, NH), 7.99 (1H, dd, *J* = 2.8 Hz, 1.2 Hz, H3), 7.75–7.69 (2H, m, H2', H6'), 7.42 (1H, dd, *J* = 8.8 Hz, 2.8 Hz, H5), 7.34 (1H, dd, *J* = 8.8 Hz, 1.2 Hz, H6), 7.09–7.03 (2H, m, H3', H5'), 4.27–4.14 (4H, m, CH_A_H_B_), 1.30 (6H, dt, *J* = 7.0 Hz, 1.0 Hz, CH_3_); ^13^C-NMR: δ 161.4, 159.5 (1C, d, *J* = 242.6 Hz, C4'), 146.1 (1C, d, *J* = 8.5 Hz), 133.9 (1C, d, *J* = 2.8 Hz, C1'), 132.2 (1C, d, *J* = 1.5 Hz), 131.5, 128.5 (1C, d, *J* = 6.1 Hz), 122.6 (1C, d, *J* = 2.9 Hz), 121.7 (2C, d, *J* = 7.8 Hz, C2', C6'), 115.6 (2C, d, *J* = 22.3 Hz, C3', C5'), 65.9 (2C, d, *J* = 5.6 Hz, CH_A_H_B_), 15.8 (2C, d, *J* = 7.4 Hz, CH_3_); ^31^P-NMR: δ 62.7. Anal. Calcd. for C_17_H_18_ClFNO_4_PS (417.82): C, 48.87; H, 4.34; N, 3.35. Found: C, 48.98; H, 4.50; N, 3.19.

*O-(4-Chloro-2-{[3-(trifluoromethyl)phenyl]carbamoyl}phenyl) O,O-diethyl phosphorothioate* (**1q**). Yield: 67%, white solid; m.p. 49–51 °C; IR: 1678 (amide I), 1606 (ν CC_aromatic_), 1564 (amide II), 1450 (ν CC_aromatic_) cm^−1^; ^1^H-NMR: δ 9.22 (1H, bs, NH), 8.06–8.02 (2H, m, H3, H2'), 7.98 (1H, d, *J* = 8.0 Hz, H6'), 7.49 (1H, t, *J* = 8.0 Hz, H5'), 7.45 (1H, dd, *J* = 8.8 Hz, 2.8 Hz, H5), 7.40 (1H, d, *J* = 8.0 Hz, H4'), 7.37 (1H, dd, *J* = 8.8 Hz, 1.3 Hz, H6), 4.29–4.16 (4H, m, CH_A_H_B_), 1.31 (6H, t, *J* = 7.0 Hz, CH_3_); ^13^C-NMR: δ 161.7, 146.2 (1C, d, *J* = 8.4 Hz), 138.4, 132.5 (1C, d, *J* = 1.6 Hz), 131.7 (1C, d, *J* = 1.1 Hz), 131.6 (1C, d, *J* = 1.9 Hz), 131.4 (1C, q, *J* = 32.3 Hz, C3'), 129.6, 128.0 (1C, d, *J* = 6.1 Hz), 123.8 (1C, q, *J* = 270.9 Hz, CF_3_), 123.1 (1C, d, *J* = 0.9 Hz), 122.6 (1C, d, *J* = 2.9 Hz), 121.1 (1C, q, *J* = 3.8 Hz, C2'), 116.8 (1C, q, *J* = 3.9 Hz, C4'), 66.0 (2C, d, *J* = 5.5 Hz, CH_A_H_B_), 15.8 (2C, d, *J* = 7.4 Hz, CH_3_); ^31^P-NMR: δ 62.7. Anal. Calcd for C_18_H_18_ClF_3_NO_4_PS (467.83): C, 46.21; H, 3.88; N, 2.99. Found: C, 45.98; H, 4.04; N, 3.19.

*O-(4-Chloro-2-{[4-(trifluoromethyl)phenyl]carbamoyl}phenyl) O,O-diethyl phosphorothioate* (**1r**). Yield: 93%, white solid; m.p. 81–82 °C; IR: 1684 (amide I), 1603 (ν CC_aromatic_), 1540 (amide II), 1475 (ν CC_aromatic_) cm^−1^; ^1^H-NMR: δ 9.24 (1H, bs, NH), 8.02 (1H, dd, *J* = 2.8 Hz, 1.2 Hz, H3), 7.89 (2H, d, *J* = 8.5 Hz, H3', H5'), 7.62 (2H, d, *J* = 8.5 Hz, H2', H6'), 7.45 (1H, dd, *J* = 8.8 Hz, 2.8 Hz, H5), 7.35 (1H, dd, *J* = 8.8 Hz, 1.3 Hz, H6), 4.28–4.16 (4H, m, CH_A_H_B_), 1.31 (6H, td, *J* = 7.0 Hz, 1.0 Hz, CH_3_); ^13^C-NMR: δ 161.8, 146.1 (1C, d, *J* = 8.7 Hz), 140.9, 132.6 (1C, d, *J* = 1.8 Hz), 131.7 (1C, d, *J* = 1.0 Hz), 131.6 (1C, d, *J* = 2.6 Hz), 128.1 (1C, d, *J* = 6.3 Hz), 126.3 (1C, q, *J* = 32.6 Hz, C4'), 126.2 (2C, q, *J* = 3.9 Hz, C3', C5'), 124.0 (1C, q, *J* = 270.0 Hz, CF_3_), 122.6 (1C, d, *J* = 3.0 Hz), 119.7, 66.0 (2C, d, *J* = 5.6 Hz, CH_A_H_B_), 15.8 (2C, d, *J* = 7.4 Hz, CH_3_); ^31^P-NMR: δ 62.6. Anal. Calcd for C_18_H_18_ClF_3_NO_4_PS (467.83): C, 46.21; H, 3.88; N, 2.99. Found: C, 46.32; H, 3.93; N, 3.20.

*O-{5-Chloro-2-[(3-chlorophenyl)carbamoyl]phenyl} O,O-diethyl phosphorothioate* (**1s**). Yield: 79%, semisolid; IR: 1660 (amide I), 1593 (ν CC_aromatic_), 1532 (amide II), 1482 (ν CC_aromatic_) cm^−1^; ^1^H-NMR: δ 9.06 (1H, bs, NH), 7.98 (1H, d, *J* = 8.5 Hz, H3), 7.87 (1H, t, *J* = 2.0 Hz, H2'), 7.58 (1H, dd, *J* = 8.3 Hz, 1.8 Hz, H6'), 7.40 (1H, t, *J* = 1.5 Hz, H6), 7.32 (1H, td, *J* = 8.5 Hz, 1.0 Hz, H4), 7.26 (1H, t, *J* = 8.0 Hz, H5'), 7.12–7.08 (1H, m, H4'), 4.28–4.17 (4H, m, CH_A_H_B_), 1.32 (6H, t, *J* = 7.0 Hz, CH_3_); ^13^C-NMR: δ 161.9, 147.9 (1C, d, *J* = 8.5 Hz), 139.1, 138.0 (1C, d, *J* = 1.9 Hz), 134.6, 132.9, 129.9, 126.3 (1C, d, *J* = 1.1 Hz), 125.2 (1C, d, *J* = 6.0 Hz), 124.5, 121.5 (1C, d, *J* = 3.0 Hz), 120.1, 118.0, 66.1 (2C, d, *J* = 5.4 Hz, CH_A_H_B_), 15.8 (2C, d, *J* = 7.4 Hz, CH_3_); ^31^P-NMR: δ 62.4. Anal. Calcd for C_17_H_18_Cl_2_NO_4_PS (434.27): C, 47.02; H, 4.18; N, 3.23. Found: C, 46.91; H, 4.29; N, 3.34.

*O-{5-Chloro-2-[(4-chlorophenyl)carbamoyl]phenyl} O,O-diethyl phosphorothioate* (**1t**). Yield: 91%, white solid; m.p. 67–68 °C (acetone-hexane); IR: 1658 (amide I), 1604, 1546 (ν CC_aromatic_), 1546 (amide II), 1492 (ν CC_aromatic_) cm^−1^; ^1^H-NMR: δ 9.07 (1H, bs, NH), 7.99 (1H, d, *J* = 8.5 Hz, H3), 7.74–7.69 (2H, m, H2', H6'), 7.40 (1H, d, *J* = 1.8 Hz, H6), 7.34–7.29 (3H, m, H4, H3', H5'), 4.29–4.17 (4H, m, CH_A_H_B_), 1.32 (6H, dt, *J* = 7.0 Hz, 1.0 Hz, CH_3_); ^13^C-NMR: δ 161.9, 147.9 (1C, d, *J* = 8.8 Hz), 137.9 (1C, d, *J* = 1.6 Hz), 136.6, 132.8, 129.4, 129.0, 126.3, 125.4 (1C, d, *J* = 6.1 Hz), 121.5 (1C, d, *J* = 2.6 Hz), 121.2, 66.1 (2C, d, *J* = 5.5 Hz, CH_A_H_B_), 15.8 (2C, d, *J* = 7.4 Hz, CH_3_); ^31^P-NMR: δ 62.3. Anal. Calcd for C_17_H_18_Cl_2_NO_4_PS (434.27): C, 47.02; H, 4.18; N, 3.23. Found: C, 47.20; H, 4.02; N, 3.10.

*O-{5-Chloro-2-[(3,4-dichlorophenyl)carbamoyl]phenyl} O,O-diethyl phosphorothioate* (**1u**). Yield: 91%, white solid; m.p. 70–71 °C; IR: 1650 (amide I), 1599, 1587 (ν CC_aromatic_), 1533 (amide II), 1472 (ν CC_aromatic_) cm^−1^; ^1^H-NMR: δ 9.14 (1H, bs, NH), 8.02 (1H, d, *J* = 2.5 Hz, H2'), 8.00 (1H, dd, *J* = 8.5 Hz, 1.0 Hz, H3), 7.58 (1H, dd, *J* = 8.8 Hz, 2.3 Hz, H6'), 7.42–7.39 (2H, m, H6, H5'), 7.32 (1H, td, *J* = 8.5 Hz, 1.0 Hz, H4), 4.31–4.18 (4H, m, CH_A_H_B_), 1.34 (6H, dt, *J* = 7.0 Hz, 1.0 Hz, CH_3_); ^13^C-NMR: δ 161.9, 148.0 (1C, d, *J* = 8.8 Hz), 138.2 (1C, d, *J* = 1.5 Hz), 137.5, 132.9, 132.8, 130.5, 127.6, 126.3, 124.9 (1C, d, *J* = 6.0 Hz), 121.7, 121.5 (1C, d, *J* = 2.6 Hz), 119.3, 66.2 (2C, d, *J* = 5.5 Hz, CH_A_H_B_), 15.8 (2C, d, *J* = 7.3 Hz, CH_3_); ^31^P-NMR: δ 62.3. Anal. Calcd for C_17_H_17_Cl_3_NO_4_PS (468.72): C, 43.56; H, 3.66; N, 2.99. Found: C, 43.69; H, 3.90; N, 3.18.

*O-{2-[(3-Bromophenyl)carbamoyl]-5-chlorophenyl} O,O-diethyl phosphorothioate* (**1v**). Yield: 49%, colour liquid; IR: 1681 (amide I), 1589 (ν CC_aromatic_), 1532 (amide II), 1479, 1420 (ν CC_aromatic_) cm^−1^; ^1^H-NMR: δ 9.07 (1H, bs, NH), 8.05–8.96 (2H, m, H3, H2΄), 7.65 (1H, d, *J* = 7.5 Hz, H6'), 7.41 (1H, s, H6), 7.34–7.18 (3H, m, H4, H4', H5'), 4.33–4.16 (4H, m, CH_A_H_B_), 1.33 (6H, t, *J* = 7.0 Hz, CH_3_); ^13^C-NMR: δ 161.9, 147.9 (1C, d, *J* = 8.5 Hz), 139.2, 138.0 (1C, d, *J* = 1.9 Hz), 132.9, 130.2, 127.4, 126.3, 125.2 (1C, d, *J* = 6.0 Hz), 122.9, 122.6, 121.5 (1C, d, *J* = 2.4 Hz), 118.5, 66.1 (2C, d, *J* = 5.4 Hz, CH_A_H_B_), 15.8 (2C, d, *J* = 7.4 Hz, CH_3_); ^31^P-NMR: δ 62.4. Anal. Calcd for C_17_H_18_BrClNO_4_PS (478.72): C, 42.65; H, 3.79; N, 2.93. Found: C, 42.54; H, 4.00; N, 3.01.

*O-{2-[(4-Bromophenyl)carbamoyl]-5-chlorophenyl} O,O-diethyl phosphorothioate* (**1w**). Yield: 93%, white solid; m.p. 80–82 °C; IR: 1681 (amide I), 1649, 1600, 1589 (ν CC_aromatic_), 1529 (amide II), 1488 (ν CC_aromatic_) cm^−1^; ^1^H-NMR: δ 9.06 (1H, bs, NH), 7.99 (1H, d, *J* = 8.5 Hz, H3), 7.68–7.64 (2H, m, H2', H6'), 7.49–7.44 (2H, m, H3', H5'), 7.40 (1H, t, *J* = 1.8 Hz, H6), 7.31 (1H, td, *J* = 8.5 Hz, 1.0 Hz, H4), 4.29–4.17 (4H, m, CH_A_H_B_), 1.33 (6H, tt, *J* = 7.1 Hz, 0.8 Hz, CH_3_); ^13^C-NMR: δ 161.9, 147.9 (1C, d, *J* = 8.8 Hz), 138.0 (1C, d, *J* = 1.4 Hz), 137.1, 132.9, 131.9, 126.3 (1C, d, *J* = 1.4 Hz), 125.4 (1C, d, *J* = 6.1 Hz), 121.6, 121.5 (1C, d, *J* = 2.8 Hz), 117.1, 66.1 (2C, d, *J* = 5.4 Hz, CH_A_H_B_), 15.8 (2C, d, *J* = 7.4 Hz, CH_3_); ^31^P-NMR: δ 62.3. Anal. Calcd for C_17_H_18_BrClNO_4_PS (478.72): C, 42.65; H, 3.79; N, 2.93. Found: C, 42.77; H, 3.84; N, 2.78.

*O-{5-Chloro-2-[(3-fluorophenyl)carbamoyl]phenyl} O,O-diethyl phosphorothioate* (**1x**). Yield: 48%, oily liquid; IR: 1662 (amide I), 1599 (ν CC_aromatic_), 1541 (amide II), 1491 (ν CC_aromatic_) cm^−1^; ^1^H-NMR: δ 9.11 (1H, bs, NH), 7.99 (1H, dd, *J* = 8.5 Hz, 1.0 Hz, H3), 7.71 (1H, dt, *J* = 11.0 Hz, 2.0 Hz, H6'), 7.42–7.26 (4H, m, H4, H6, H2', H5'), 6.84 (1H, td, *J* = 8.5 Hz, 1.8 Hz, H4'), 4.30–4.18 (4H, m, CH_A_H_B_), 1.32 (6H, dt, *J* = 7.0 Hz, 0.9 Hz, CH_3_); ^13^C-NMR: δ 162.9 (1C, d, *J* = 243.3 Hz, C3'), 162.0, 147.9 (1C, d, *J* = 8.6 Hz), 139.4 (1C, d, *J* = 10.9 Hz), 138.0 (1C, d, *J* = 1.9 Hz), 132.9, 130.0 (1C, d, *J* = 9.4 Hz), 126.3 (1C, d, *J* = 1.4 Hz), 125.3 (1C, d, *J* = 6.1 Hz), 121.5 (1C, d, *J* = 3.0 Hz), 115.3 (1C, d, *J* = 3.0 Hz, C6'), 111.2 (1C, d, *J* = 21.3 Hz), 107.5 (1C, d, *J* = 26.4 Hz), 66.1 (2C, d, *J* = 5.5 Hz, CH_A_H_B_), 15.8 (2C, d, *J* = 7.3 Hz, CH_3_); ^31^P-NMR: δ 62.4. Anal. Calcd for C_17_H_18_ClFNO_4_PS (417.82): C, C, 48.87; H, 4.34; N, 3.35. Found: C, 49.02; H, 4.50; N, 3.24.

*O-{5-Chloro-2-[(4-fluorophenyl)carbamoyl]phenyl} O,O-diethyl phosphorothioate* (**1y**). Yield: 83%, white solid; m.p. 41–42 °C; IR: 1655 (amide I), 1640, 1616 (ν CC_aromatic_), 1560 (amide II), 1507, 1484 (ν CC_aromatic_) cm^−1^; ^1^H-NMR: δ 9.02 (1H, bs, NH), 7.99 (1H, dd, *J* = 8.8 Hz, 1.0 Hz, H3), 7.75–7.69 (2H, m, H2', H6'), 7.40 (1H, t, *J* = 1.8 Hz, H6), 7.31 (1H, ddd, *J* = 8.8 Hz, 2.0 Hz, 1.0 Hz, H4), 7.08–7.02 (2H, m, H3', H5'), 4.29–4.17 (4H, m, CH_A_H_B_), 1.32 (6H, dt, *J* = 7.0 Hz, 0.9 Hz, CH_3_); ^13^C-NMR: δ 161.8, 159.4 (1C, d, *J* = 242.5 Hz, C4'), 147.9 (1C, d, *J* = 8.9 Hz), 137.8 (1C, d, *J* = 1.9 Hz), 134.0 (1C, d, *J* = 2.8 Hz, C1'), 132.8, 126.2 (1C, d, *J* = 1.4 Hz), 125.5 (1C, d, *J* = 6.1 Hz), 121.7 (2C, d, *J* = 7.8 Hz, C2', C6'), 121.5 (1C, d, *J* = 3.0 Hz), 115.6 (2C, d, *J* = 21.2 Hz, C3', C5'), 66.0 (2C, d, *J* = 5.6 Hz, CH_A_H_B_), 15.8 (2C, d, *J* = 7.4 Hz, CH_3_); ^31^P-NMR: δ 62.4. Anal. Calcd for C_17_H_18_ClFNO_4_PS (417.82): C, 48.87; H, 4.34; N, 3.35. Found: C, 48.66; H, 4.30; N, 3.18.

*O-(5-Chloro-2-{[3-(trifluoromethyl)phenyl]carbamoyl}phenyl) O,O-diethyl phosphorothioate* (**1z**). Yield: 78%, oily liquid; IR: 1681 (amide I), 1598 (ν CC_aromatic_), 1552 (amide II), 1493, 1446 (ν CC_aromatic_) cm^−1^; ^1^H-NMR: δ 9.22 (1H, bs, NH), 8.07 (1H, s, H2'), 8.01 (1H, dd, *J* = 8.8 Hz, 1.0 Hz, H3), 7.96 (1H, d, *J* = 8.0 Hz, H6'), 7.48 (1H, t, *J* = 7.8 Hz, H5'), 7.43 (1H, t, *J* = 1.5 Hz, H6), 7.39 (1H, d, *J* = 8.0 Hz, H4'), 7.32 (1H, ddd, *J* = 8.5 Hz, 1.8 Hz, 1.0 Hz, H4), 4.30–4.18 (4H, m, CH_A_H_B_), 1.32 (6H, dt, *J* = 7.0 Hz, 1.0 Hz, CH_3_); ^13^C-NMR: δ 162.0, 148.0 (1C, d, *J* = 8.6 Hz), 138.5, 138.2 (1C, d, *J* = 2.0 Hz), 132.9, 131.3 (1C, q, *J* = 32.3 Hz, C3'), 129.5, 126.3 (1C, d, *J* = 1.4 Hz), 125.0 (1C, d, *J* = 6.1 Hz), 123.8 (1C, q, *J* = 270.9 Hz, CF_3_), 123.1 (1C, d, *J* = 0.9 Hz), 121.5 (1C, d, *J* = 3.1 Hz), 121.0 (1C, q, *J* = 3.8 Hz, C2'), 116.7 (1C, q, *J* = 4.0 Hz, C4'), 66.1 (2C, d, *J* = 5.5 Hz, CH_A_H_B_), 15.7 (2C, d, *J* = 7.4 Hz, CH_3_); ^31^P-NMR: δ 62.5. Anal. Calcd for C_18_H_18_ClF_3_NO_4_PS (467.83): C, 46.21; H, 3.88; N, 2.99. Found: C, 46.37; H, 3.75; N, 3.13.

*O-(5-Chloro-2-{[4-(trifluoromethyl)phenyl]carbamoyl}phenyl) O,O-diethyl phosphorothioate* (**1zz**). Yield: 78%, white solid; m.p. 61–62 °C (acetone-hexane); IR: 1668 (amide I), 1594 (ν CC_aromatic_), 1542 (amide II), 1474 (ν CC_aromatic_) cm^−1^; ^1^H-NMR: δ 9.25 (1H, bs, NH), 8.01 (1H, dd, *J* = 8.7 Hz, 0.9 Hz, H3), 7.89 (2H, d, *J* = 8.4 Hz, H3', H5'), 7.62 (2H, d, *J* = 8.4 Hz, H2', H6'), 7.42 (1H, t, *J* = 1.8 Hz, H6), 7.31 (1H, ddd, *J* = 8.6 Hz, 2.0 Hz, 1.0 Hz, H4), 4.33–4.15 (4H, m, CH_A_H_B_), 1.32 (6H, dt, *J* = 7.0 Hz, *J* = 0.9 Hz, CH_3_); ^13^C-NMR: δ 162.1, 148.0 (1C, d, *J* = 8.9 Hz), 141.0, 138.2 (1C, d, *J* = 2.0 Hz), 132.9 (1C, d, *J* = 1.0 Hz), 126.3 (1C, d, *J* = 1.5 Hz), 125.1 (1C, d, *J* = 6.1 Hz), 126.2 (2C, q, *J* = 3.8 Hz, C3', C5'), 126.2 (1C, q, *J* = 32.5 Hz, C4'), 124.1 (1C, q, *J* = 269.9 Hz, CF_3_), 121.5 (1C, d, *J* = 3.0 Hz), 119.7, 66.1 (2C, d, *J* = 5.6 Hz, CH_A_H_B_), 15.8 (2C, d, *J* = 7.4 Hz, CH_3_); ^31^P-NMR: δ 62.3. Anal. Calcd for C_18_H_18_ClF_3_NO_4_PS (467.83): C, 46.21; H, 3.88; N, 2.99. Found: C, 46.06; H, 3.71; N, 2.82.

### 3.3. In Vitro Antimycobacterial Activity

The *in vitro* antimycobacterial activity of the synthesized compounds was determined against *Mycobacterium tuberculosis* My 331/88 (H_37_Rv; dilution of strain 10^−3^), *M. avium* My 330/88 (dilution of strain 10^−5^), *M. kansasii* My 235/80 (dilution of strain 10^−4^) and *M. kansasii* 6509/96 (dilution of strain 10^−4^). All of the strains were obtained from the Czech National Collection of Type Cultures (CNCTC, Brno, Czech Republic) with the exception of *M. kansasii* 6509/96, which was clinically isolated. The antimycobacterial activity of the compounds was determined in a Šula’s semisynthetic medium (SEVAC, Prague, Czech Republic) *via* the micromethod for the determination of the minimum inhibitory concentration (MIC) at 37 °C after 14 and 21 days and after 7, 14 and 21 days for *M. kansasii* [15]. The tested compounds and *para*-aminosalicylic acid (PAS) were added to the medium in DMSO solutions, and INH was used as a standard in a sterile water solution. The concentrations of the tested compounds were used as following: 500, 250, 125, 62.5, 32, 16, 8, 4, 2, 1 and 0.5 µM. The same concentrations within the range from 0.5 to 250 µM were used for INH and PAS; these compounds were selected as comparative drugs.

### 3.4. Determination of IC_50_s for Cholinesterases

The IC_50_ values were determined using the spectrophotometric Ellman’s method, which is a simple, rapid and direct method to determine the SH and -S-S- group content in proteins [16]. This method is widely used for cholinesterase activity evaluation and screening of the efficiency of cholinesterase inhibitors. Cholinesterase activity is measured indirectly by quantifying the concentration of 5-thio-2-nitrobenzoic acid (TNB) ion formed in the reaction between the thiol reagent 5,5'-dithiobis-2-nitrobenzoic acid (DTNB) and thiocholine, a product of substrate hydrolysis by cholinesterase (*i.e.*, acetylthiocholine, ATCh) [17]. All the tested compounds were dissolved in dimethyl sulfoxide (concentration 0.01 M) and then diluted in demineralised water (concentration 0.001 M and 0.0001 M). The Ellman’s method is slightly modified according to Zdrazilova *et al.* [14]. Acetylcholinesterase was obtained from electric eel (*Electrophorus electricus* L.) and butyrylcholinesterase from equine serum.

## 4. Conclusions

A series of 27 new salicylanilide derivatives containing diethyl thiophosphate moieties was synthesized with the goal of improving the antimycobacterial activity of salicylanilide diethyl phosphates as potential salicylanilide prodrugs presented earlier. However, the expected improvement was not achieved. Derivatives of 5-bromosalicylic acid, 3,4-dichloroaniline, 3- and 4-(trifluoro-methyl)aniline exhibited superior antimycobacterial activity. The thiophosphate moiety also inspired us to evaluate this series against acetylcholinesterase and butyrylcholinesterase. All the discussed compounds expressed a significant inhibition of both AChE and BChE. Their activities were compared to those of the known acetylcholinesterase inhibitors galanthamine and rivastigmine. All tested compounds showed higher or comparable activity to that of rivastigmine and slightly lower than that of galanthamine. IC_50_ values for salicylanilide diethyl thiophosphates were found to be similar for all of the compounds and also for both AChE and BChE. We demonstrated unequivocally that salicylanilide diethyl thiophosphates share dual biological activity as inhibitors of both cholinesterases and various mycobacteria. Obviously, there is no correlation between both activities. It is a result of different expected mechanisms of action and the active part of the molecule: the salicylanilide scaffold confers antimicrobial activity preferentially, but the cholinesterase inhibition arises from the presence of the thiophosphate moiety.
